# 3,4-Dimethoxychalcone-induced caloric restriction inhibits PANoptosis to promote ischemic and diabetic skin flap survival: experimental studies

**DOI:** 10.1097/JS9.0000000000004619

**Published:** 2026-01-19

**Authors:** Gaoxiang Yu, Jiayi Zhao, Xuwei Zhu, Ningning Yang, Junsheng Lou, Zhuliu Chen, Haojie Zhang, Tafadzwa Chaire, Weiyang Gao, Yuepiao Cai, Xiangyang Wang, Jian Ding, Jian Xiao, Kailiang Zhou, Jianjun Qi

**Affiliations:** a Department of Orthopaedics, The Second Affiliated Hospital and Yuying Children’s Hospital of Wenzhou Medical University, Wenzhou, China; b Zhejiang Provincial Key Laboratory of Orthopaedics, Wenzhou, China; c The Second Clinical Medical College of Wenzhou Medical University, Wenzhou, China; d Department of Hand Microsurgery and Plastic Reconstructive Surgery, Ningbo No.6 Hospital, Ningbo, Zhejiang, China; e Molecular Pharmacology Research Center, School of Pharmaceutical Science, Wenzhou Medical University, Wenzhou, China; f Department of Laboratory Medicine, The First Affiliated Hospital of Wannan Medical College (Yijishan Hospital of Wannan Medical College), Wuhu, China

**Keywords:** 3,4-dimethoxychalcone, autophagy, diabetic flaps, ischemic flaps, miR-107-3p, PANoptosis

## Abstract

**Background:**

Ischemic necrosis frequently affects the distal portion of skin flaps, particularly in diabetic patients. PANoptosis, a form of inflammatory programmed cell death, is implicated in vascular damage. This study examined whether 3,4-dimethoxychalcone (3,4-DC), a novel caloric restriction (CR) mimetic, could inhibit PANoptosis and promote the survival of ischemic and diabetic flaps.

**Methods:**

Flap viability was evaluated using laser Doppler blood flow imaging and histological analysis. Western blotting and immunofluorescence were used to assess PANoptosis and autophagy. Quantitative PCR was used to measure microRNA levels. Caspase-1 knockout and *db/db* mice were used to explore the effects of 3,4-DC on pyroptosis and diabetic complications.

**Results:**

3,4-DC significantly improved ischemic flap survival and enhanced tissue perfusion. PANoptosis was inhibited, and autophagy was activated following 3,4-DC treatment, whereas these effects were abolished by the autophagy inhibitor chloroquine. Transcription factor EB (TFEB) inhibition reduced autophagy and reversed the protective effects of 3,4-DC. miR-107-3p was identified as a 3,4-DC-responsive microRNA that modulates TFEB nuclear translocation through the miR-107-3p/Wnt3a/mTOR pathway. Similar therapeutic effects were observed in diabetic flaps.

**Conclusion:**

3,4-DC promotes the survival of ischemic and diabetic skin flaps by activating autophagy and inhibiting PANoptosis through the miR-107-3p/Wnt3a/mTOR/TFEB axis.

## Introduction

Random-pattern skin flaps are widely used in reconstructive surgery due to their simplicity and versatility^[1]^. However, ischemic necrosis remains a leading cause of failure in skin flap transplantation, particularly at the distal end where the blood supply is compromised^[2,3]^. Enhancing flap viability under ischemic conditions is, therefore, a key clinical challenge. While antioxidant, anti-inflammatory, and proangiogenic approaches have shown benefits^[4,5]^. Apoptosis and other programmed cell death (PCD) modalities (such as pyroptosis and necroptosis) have been studied in ischemic skin flaps^[6]^, and enhancing autophagy mitigated cellular damage and improved flap survival^[7]^. However, although these mechanisms have been examined individually, the integrated concept of PANoptosis and its relationship with autophagy remain underexplored. Therefore, developing strategies to enhance distal flap perfusion and cell survival is critically important for improving flap viability.

Multiple forms of PCD contribute to ischemic injury in skin flaps. Apoptosis, pyroptosis, and necroptosis each drive cellular demise and inflammation in response to ischemic stress^[8–10]^. Traditionally, these pathways were studied independently; however, accumulating evidence indicates that they are highly interconnected rather than isolated processes. Recent studies have highlighted PANoptosis – a regulated cell death mechanism that integrates pyroptosis, apoptosis, and necroptosis via the PANoptosome complex^[11,12]^. PANoptosis is characterized by key features of pyroptosis, apoptosis, and/or necroptosis and, by definition, is not fully explained by any one of these cell death pathways alone^[13]^. The PANoptosome activates caspase-3 (CASP-3) and CASP-8 (apoptosis), (p)MLKL, (p)RIPK1, and (p)RIPK3 (necroptosis), and NLRP3, ASC, CASP-1, GSDMD, and IL-1β (pyroptosis), driving inflammation and cell death^[14,15]^. Targeting PANoptosis may allow us to affect pyroptosis, apoptosis, and necroptosis at the same time, which might be more effective than blocking only one pathway. However, the role of PANoptosis in ischemic skin flaps has not yet been defined. By contrast, autophagy is a lysosome-mediated degradation process that can remove damaged organelles and support cell survival under stress^[16]^. Balancing these pathways is crucial: enhancing protective autophagy while limiting inflammatory PCD may reduce flap necrosis.

The transcription factor EB (TFEB) is a master regulator of the autophagy–lysosome pathway, driving the expression of autophagy and lysosomal genes^[17,18]^. Ischemic injury disrupts autophagy flux in flaps, and TFEB activation may restore this balance and inhibit PANoptosis. Caloric restriction (CR) is a well-recognized intervention that extends lifespan and promotes stress resistance, largely by activating autophagy and suppressing PCD^[19]^. However, its clinical application is limited by feasibility and patient compliance^[20]^. Caloric restriction mimetics (CRMs) are a class of small molecules designed to reproduce the beneficial cellular effects of CR – such as autophagy induction, metabolic reprogramming, and anti-inflammatory activity – without requiring dietary restriction^[21]^. Notably, the CRM 3,4-dimethoxychalcone (3,4-DC), a synthetic chalcone derivative, has shown a safety profile in previous studies, as it induces TFEB nuclear translocation and lysosomal biogenesis without obvious toxicity *in vitro* or in animal models^[22]^, but it has not yet been tested in humans. Treatment with 3,4-DC enhanced TFEB-mediated autophagy and concomitantly attenuated pyroptosis and necroptosis in a spinal cord injury model^[23]^. Reduction of TFEB in this model abolished the protective effects of 3,4-DC, demonstrating that TFEB-driven autophagy is essential for limiting inflammatory cell death^[23]^. These findings suggest that pharmacological activation of TFEB and autophagy may similarly protect ischemic skin flap tissues.

Our preliminary miRNA sequencing and dual-luciferase reporter assays identified miR-107-3p as a candidate regulator that directly targets and suppresses Wnt3a. Wnt3a is a key ligand in the canonical Wnt pathway and can influence mTOR signaling to promote cell growth and survival^[24,25]^. Thus, upregulation of miR-107-3p could downregulate Wnt3a/mTOR signaling and thereby affect cell proliferation and autophagy balance. Targeting the miR-107-3p/Wnt3a/mTOR axis may represent a novel strategy to enhance cell survival in ischemic flaps by coordinating growth signals and autophagic activity.

Diabetes mellitus exacerbates flap ischemia via microvascular dysfunction and oxidative stress^[26]^. Chronic hyperglycemia leads to endothelial damage and advanced glycation end products, which impair capillary perfusion^[27]^. Indeed, diabetic animal models exhibit significantly larger areas of random-flap necrosis compared to nondiabetic controls according to our preliminary experiments. Therefore, elucidating the mechanisms that preserve flap viability in diabetic ischemic environments is essential for improving surgical outcomes in diabetic patients.

**HIGHLIGHTS**

- 3,4-DC promotes ischemic flap survival through reducing PANoptosis.
- 3,4-DC reduces PANoptosis by enhancing autophagy in ischemic flaps.
- The effect of 3,4-DC on autophagy is mediated by TFEB activation following ischemic flap.
- 3,4-DC promotes TFEB activation via miR-107-3p/wnt3a/mTOR signaling.
- 3,4-DC as a CR mimic has protective effects on diabetic flaps.

In this study, we investigated whether 3,4-DC promotes ischemic skin flap survival by inhibiting PANoptosis by autophagy and whether this effect is mediated by miR-107-3p-dependent regulation of the Wnt3a/mTOR/TFEB signaling pathway. We further evaluated the therapeutic potential of 3,4-DC in diabetic mice, in which flap failure is exacerbated. Our findings uncover a novel potential miRNA-regulated therapeutic approach to improve outcomes in ischemic and diabetic flap surgery.

## Materials and methods

All animal procedures were approved by the university ethics committee (wydw2024-0283) and followed ARRIVE guidelines^[28]^. As oestrogen is known to affect flap necrosis, only male mice were used in this study^[29]^. Random-pattern skin flaps (1.5 × 4.5 cm^2^) were created in male C57BL/6J and *db/db* mice as previously described^[5]^. Ischemia was induced by ligation of the bilateral sacral vessels. The concentration of 3,4-DC used was determined on the basis of previously published literature^[22]^, as well as preliminary efficacy and toxicity assessments conducted with our model (Supplemental Digital Content Figure S1, available at: http://links.lww.com/JS9/G576). 3,4-DC (200 mg/kg) was administered intraperitoneally every other day for 7 days. Laser Doppler blood flow imaging, histological analysis, immunofluorescence (IF), western blotting (WB), and qPCR were performed on postoperative day 7 to assess flap viability, autophagy, PANoptosis, and associated molecular changes. AAV-shTFEB and AAV-miR-107-3p were used for in vivo gene modulation. All group sizes were *n* = 5 unless otherwise stated. Further experimental details, including miRNA sequencing, luciferase reporter assays, reagents, and antibodies, are provided in the Supplemental Digital Content Methods, available at: http://links.lww.com/JS9/G590.

## Results

### 3,4-DC promoted ischemic flap survival

**Figure 1.**
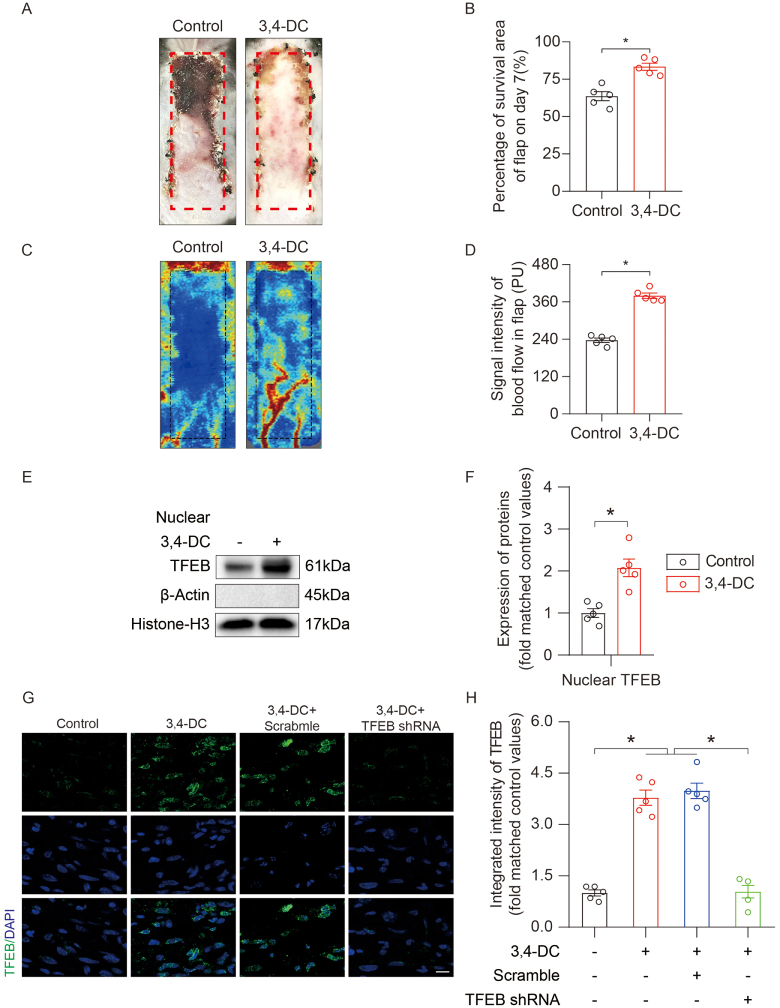
3,4-DC increased TFEB activity and promoted ischaemic skin flap survival. (A) Digital photograph of the surviving area of the flap on postoperative day (POD) 7. (B) Comparison of the survival area between the two groups on POD 7 (n = 5). (C) Subcutaneous blood flow was assessed by measuring LDBF on POD 7. (D) Comparison of the signal intensity of blood flow in ischaemic skin flaps between the two groups on POD 7 (n = 5). (E) Nuclear levels of the TFEB protein in skin extracts from the control and 3,4-DC groups. (F) Comparison of the nuclear TFEB level between the two groups on POD 7 (n = 5). Scale bars represent 10 μm. (G) IF staining for TFEB in the skin of mice treated with corn oil, 3,4-DC, 3,4-DC+scrambled, or 3,4-DC+TFEB shRNA on POD 7. Scale bars represent 10 μm. (H) Comparison of the integrated intensity of TFEB in the dermal layer among the four groups (n = 5). The error bars represent the SEMs. Significance: *P < 0.05, significantly different, as indicated; ns, not significant; two-tailed, unpaired *t*-test; ANOVA with the LSD post hoc analysis (equal variances of the groups) or Dunnett’s T3 method (unequal variances of the groups).

The toxicity and therapeutic efficacy of 3,4-DC were evaluated in mice. No significant changes in body weight or serum ALT/AST levels were observed across the dosage groups (0–300 mg/kg) (Supplemental Digital Content Figure S1A and B, available at: http://links.lww.com/JS9/G576), and although the serum creatinine levels increased slightly at 250–300 mg/kg, they remained within the normal range (Supplemental Digital Content Figure S1C, available at: http://links.lww.com/JS9/G576). Hematoxylin–eosin (HE) staining revealed no pathological changes in the liver, kidney, or heart, confirming the absence of organ toxicity (Supplemental Digital Content Figure S1D, available at: http://links.lww.com/JS9/G576). Among the tested doses, 200 mg/kg was identified as optimal, significantly improving flap survival (83.37 ± 5.40 vs. 63.64 ± 6.75%, *P* < 0.001), enhancing blood perfusion (LDBF, *P* < 0.001) (Supplemental Digital Content Figure S1E, available at: http://links.lww.com/JS9/G576, and Fig. 1A–D). At this dose, urinary ketone levels (β-hydroxybutyrate) and AMPK/SIRT1 activity were elevated, suggesting a CR-mimicking metabolic state (Supplemental Digital Content Figure S1G and H, available at: http://links.lww.com/JS9/G576). However, there was no significant change in the food intake of the two groups of mice (Supplemental Digital Content Figure S1F, available at: http://links.lww.com/JS9/G576, *P* = 0.44). In addition, we compared flap outcomes between continuous administration of 3,4-DC for 7–14 days and early discontinuation after day 7 (Supplemental Digital Content Figure S1I, available at: http://links.lww.com/JS9/G576). No significant difference was observed between the groups, suggesting that drug withdrawal did not lead to further necrosis progression. HE staining revealed no discernible pathological changes in the heart, liver, or kidneys after 2 weeks of 3,4-DC administration (Supplemental Digital Content Figure S1E, available at: http://links.lww.com/JS9/G576). HE staining and IF further revealed increased angiogenesis and CD34/ACTA2-positive vessels in area II of the flaps on POD7 (Supplemental Digital Content Figure S2A–D, available at: http://links.lww.com/JS9/G576). These findings indicate that 3,4-DC is nontoxic and effectively promotes ischemic skin flap survival.

**Figure 2.**
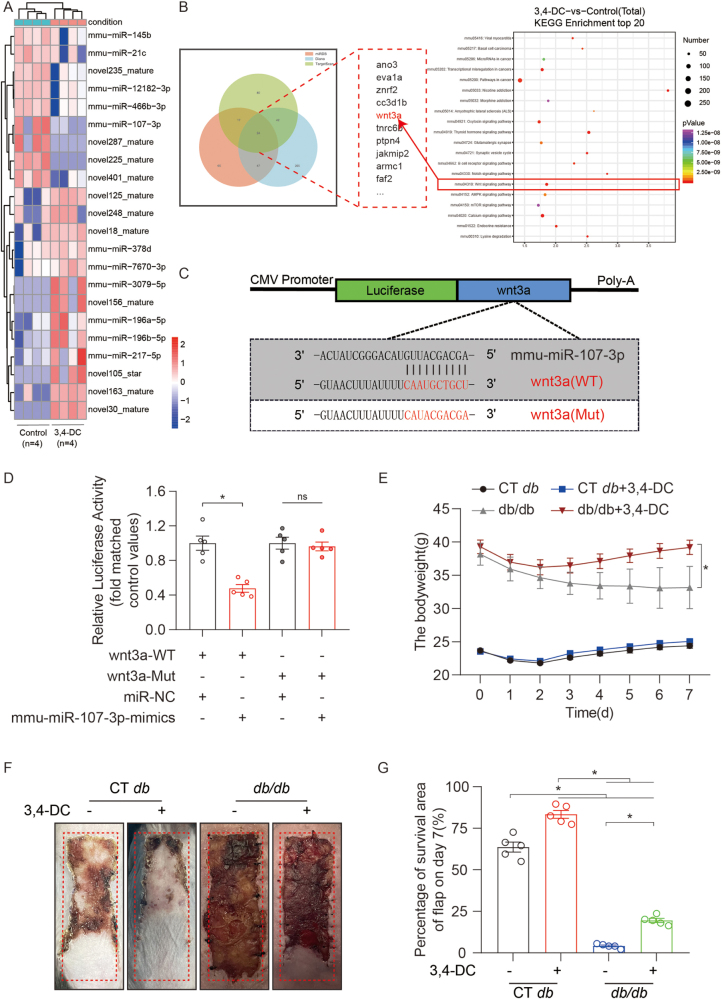
3,4-DC decreased miR-107-3p and promoted diabetic skin flap survival. (A) Heatmap showing differentially expressed miRs between the flaps of mice treated with corn oil and 3,4-DC on POD 7. (B) Schematic illustration showing the overlap of the target genes of mmu-miR-107-3p, as predicted by miRDB, TargetScan and DIANA. The top 20 KEGG pathways associated with the target genes of the miRs. (C-D) Luciferase reporter assay and comparison of the relative luciferase activity in 293T cells transfected with *Wnt3a*-WT, *Wnt3a*-MUT, *miR-NC* or *mmu-miR-107-3p* mimic for 48 h (n = 5). (E) Body weights of the mice in the *WT/db*, *WT/db*+3,4-DC, *db/db* and *db/db*+3,4-DC groups on POD 7. (F) Digital photograph of the surviving area of the flap on POD 7. (G) Comparison of the surviving areas among the four groups on POD 7 (n = 5). The error bars represent the SEMs. Significance: *p < 0.05, significantly different, as indicated; ns, not significant; ANOVA with the LSD post hoc analysis (equal variances of the groups) or Dunnett’s T3 method (unequal variances of the groups).

### 3,4-DC inhibited PANoptosis in ischemic flaps

PANoptosis is an integrated cell-death process involving apoptosis, necroptosis, and pyroptosis^[30]^. To investigate the effects of 3,4-DC on cell death, markers of apoptosis, pyroptosis, and necroptosis were measured in the flap tissues. Strong evidence for the connection between pyroptosis and flap necrosis was not provided in a previous study^[10]^. Therefore, CASP-1 KO mice were used to verify whether 3,4-DC affects pyroptosis to reduce skin flap necrosis. IF staining of the flap sections revealed that 3,4-DC markedly reduced signals for apoptotic (CASP-3), pyroptotic (NLRP3, ASC, CASP-1, GSDMD, IL-1β, and IL-18), and necroptotic ((p)MLKL, (p)RIPK1, and (p)RIPK3) markers (Supplemental Digital Content Figure S3A, C, and E, available at: http://links.lww.com/JS9/G576). WB analysis confirmed that 3,4-DC significantly suppressed the ischemia-induced increases in the levels of apoptotic-, pyroptotic-, and necroptotic-related proteins (Supplemental Digital Content Figure S3B, D, and F, available at: http://links.lww.com/JS9/G576). These findings indicate that 3,4-DC inhibits PANoptosis in ischemic flaps.

### 3,4-DC inhibited PANoptosis and facilitated ischemic flap survival by increasing autophagy

Because autophagy counteracted PCD, we measured autophagic flux in the flaps^[18]^. During flap ischemia, autophagy is impaired, but 3,4-DC significantly enhances autophagy flux, as evidenced by increased LC3, CTSD, LAMP2, VPS34, Beclin1, mat/pro-CTSL, mat/pro-CTSD, mat/pro-CTSB, and LC3II levels, along with decreased p62 expression (Supplemental Digital Content Figure S4A–F, available at: http://links.lww.com/JS9/G576). To verify the role of autophagy in flap survival, chloroquine (CQ) was used to block autophagosome–lysosome fusion. In the 3,4-DC + CQ group, CTSD levels decreased, whereas LC3 and p62 levels increased, indicating autophagy inhibition (Supplemental Digital Content Figure S5A and B, available at: http://links.lww.com/JS9/G576). CQ reversed the protective effects of 3,4-DC, with increased PANoptosis markers (Supplemental Digital Content Figure S5C–H, available at: http://links.lww.com/JS9/G576), reduced flap survival (58.31 ± 7.13 vs. 83.37 ± 5.40%, *P* < 0.001), decreased blood flow, reduced angiogenesis, and fewer CD34/ACTA2-positive vessels (Supplemental Digital Content Figure S6A–H, available at: http://links.lww.com/JS9/G576). These results confirm that 3,4-DC promotes ischemic flap survival by enhancing autophagy and inhibiting PANoptosis.

### 3,4-DC facilitated ischemic flaps by increasing TFEB translocation in ischemic flaps

TFEB is a master regulator of the autophagy-lysosome pathway^[31]^. The role of 3,4-DC in promoting autophagy via TFEB translocation was investigated^[23,32]^. In the 3,4-DC group, nuclear TFEB expression and the percentage of TFEB-positive nuclei were significantly elevated (Fig. 1E-H), indicating increased TFEB translocation. TFEB shRNA knockdown markedly reduced nuclear TFEB expression and reversed 3,4-DC-induced translocation (Fig. 1G-H and Supplemental Digital Content Figure S7A, available at: http://links.lww.com/JS9/G576). Consequently, the LC3 and CTSD expression levels decreased in the 3,4-DC + TFEB shRNA group, and WB confirmed the reversal of autophagy activation (Supplemental Digital Content Figure S7B and C, available at: http://links.lww.com/JS9/G576). Moreover, TFEB silencing counteracted the inhibitory effects of 3,4-DC on apoptosis, necroptosis, and pyroptosis (Supplemental Digital Content Figures S7D–G and S8A–D, available at: http://links.lww.com/JS9/G576), leading to reduced flap survival (61.75 ± 3.53 vs. 83.37 ± 5.40%, *P* < 0.001), decreased blood flow, reduced angiogenesis, and fewer CD34/ACTA2-positive vessels (Supplemental Digital Content Figure S9A–H, available at: http://links.lww.com/JS9/G576). These findings indicate that 3,4-DC promotes ischemic flap survival by enhancing TFEB nuclear translocation and autophagy.

### 3,4-DC enhanced TFEB translocation by downregulating miR-107-3p in ischemic flaps

To identify upstream regulators of TFEB, we performed miRNA profiling. Small RNA sequencing identified several differentially expressed miRNAs in flaps treated with 3,4-DC, among which miR-107-3p emerged as a key target because of its significant upregulation and relatively high expression level. Although miR-196-5p was also upregulated, our previous RNA-seq data demonstrated that its expression change was not specific to 3,4-DC treatment, as it was differentially expressed in both ischemic and nonischemic flaps^[33]^. In contrast, miR-107-3p expression was specifically modulated by 3,4-DC (Fig. 2A and Supplemental Digital Content Figure S10A, available at: http://links.lww.com/JS9/G576), supporting its role as a critical mediator in this context. Therefore, miR-107-3p was prioritized for further mechanistic investigation. qPCR confirmed a significant reduction in mmu-miR-107-3p expression following 3,4-DC treatment. To evaluate its functional role, AAV-miR-107-3p was used to overexpress miR-107-3p in treated flaps (Supplemental Digital Content Figure S10B, available at: http://links.lww.com/JS9/G576). Overexpression reversed the 3,4-DC-induced increase in nuclear TFEB, autophagy activation, and PANoptosis inhibition, as shown by WB and IF (Supplemental Digital Content Figures S10C and S11A–G, available at: http://links.lww.com/JS9/G576). Moreover, compared with 3,4-DC and the scramble control, AAV-miR-107-3p reduced flap survival (69.90 ± 5.29 vs. 83.37 ± 5.40%, *P* < 0.005), blood perfusion, and angiogenesis (Supplemental Digital Content Figure S12A–H, available at: http://links.lww.com/JS9/G576). These results indicate that 3,4-DC promotes autophagy, inhibits PANoptosis, and improves ischemic flap survival via downregulation of miR-107-3p and subsequent TFEB activation.

### 3,4-DC activated TFEB via the miR-107-3p/Wnt3a/mTOR signaling pathway in ischemic flaps

Positive regulation of miR-107-3p was found to inhibit the therapeutic effects of 3,4-DC. Target gene prediction using miRDB, DIANA, and TargetScan identified 54 overlapping genes, with KEGG analysis highlighting the Wnt signaling pathway and Wnt3a as key targets (Fig. 2B). Dual-luciferase assays confirmed that miR-107-3p directly targets Wnt3a (Fig. 2C and D). Given that 3,4-DC activates TFEB via mTOR inhibition^[23]^ and that Wnt3a promotes autophagy by suppressing mTOR through β-catenin activation^[34–36]^, we investigated the miR-107-3p/Wnt3a/mTOR axis. WB results revealed that 3,4-DC upregulated Wnt3a and β-catenin and inhibited p-mTOR (Supplemental Digital Content Figure S13A and B, available at: http://links.lww.com/JS9/G576), and these effects were reversed by AAV-miR-107-3p (Supplemental Digital Content Figure S13C and D, available at: http://links.lww.com/JS9/G576), confirming that Wnt3a is a functional miR-107-3p target. Furthermore, the Wnt3a inhibitor IWP-2 and the mTOR agonist MHY1485 reversed 3,4-DC-induced TFEB nuclear translocation, autophagy activation, PANoptosis suppression (Supplemental Digital Content Figure S14A–C, available at: http://links.lww.com/JS9/G576), and flap survival, angiogenesis, and perfusion (Supplemental Digital Content Figure S15A–H, available at: http://links.lww.com/JS9/G576). These results demonstrate that 3,4-DC promotes TFEB activation and enhances ischemic flap survival through the miR-107-3p/Wnt3a/mTOR signaling pathway.

### 3,4-DC enhances autophagy, inhibits PANoptosis and promotes flap survival in diabetic mice

Finally, given that diabetes increases the risk of flap graft failure^[37]^, we investigated whether 3,4-DC could improve ischemic flap survival in diabetic (*db/db*) mice by modulating PANoptosis. Postoperative weight loss was observed in all groups, with *db/db* mice showing the poorest recovery (Fig. 2E). Flap necrosis was extensive in the *db/db* group but was partially rescued by 3,4-DC (19.55 ± 2.74 vs. 4.18 ± 1.29%, *P* < 0.001) (Fig. 2F and G). LDBF analysis revealed impaired perfusion in *db/db* mice, which was partly improved by 3,4-DC (Supplemental Digital Content Figure S16B and C, available at: http://links.lww.com/JS9/G576). qPCR revealed that 3,4-DC reduced miR-107-3p levels in *db/db* mice (Supplemental Digital Content Figure S16A, available at: http://links.lww.com/JS9/G576). WB analysis revealed that 3,4-DC treatment restored Wnt3a, β-catenin, and nuclear TFEB expression while inhibiting p-mTOR, which was consistent with previous findings (Supplemental Digital Content Figure S16D–G, available at: http://links.lww.com/JS9/G576). Together, these results suggest that 3,4-DC promotes autophagy, suppresses PANoptosis, and enhances diabetic flap survival via the miR-107-3p/Wnt3a/mTOR/TFEB signaling pathway.

## Discussion

Random-pattern skin flaps remain a cornerstone of reconstructive surgery, yet their clinical utility is limited by distal ischemic necrosis^[9,38]^. Consistent with our previous reports, disrupted autophagy flux contributes critically to ischemic flap failure^[33]^. 3,4-DC, a newly identified CRM, was shown here to significantly improve flap survival by restoring autophagy and suppressing inflammatory cell death^[23]^.

Mechanistically, 3,4-DC inhibited PANoptosis – a synergistic form of PCD encompassing pyroptosis, apoptosis, and necroptosis – via activation of the miR-107-3p/Wnt3a/mTOR/TFEB signaling pathway. PANoptosis, driven by PANoptosome-mediated release of CASP-8, RIPK3, and NLRP3, is increasingly recognized in ischemic and inflammatory conditions. By downregulating key effectors across these pathways, 3,4-DC attenuated excessive cell death in flap tissue.

Our results further demonstrated that 3,4-DC promotes the nuclear translocation of TFEB, a transcriptional regulator of lysosomal and autophagic genes, thereby increasing autophagic flux. Inhibition of TFEB or blockade of autophagy reversed the cytoprotective effects of 3,4-DC and restored PANoptosis activation, highlighting a causal relationship. We recognize that autophagy is a double-edged sword, as excessive activation may lead to cell death in certain contexts, such as prolonged ischemia. In this study, the dose of 3,4-DC used was carefully selected on the basis of dose‒response assessments to ensure efficacy without inducing autophagy-related toxicity. Within the applied concentration and 7-day treatment period, no signs of maladaptive autophagy or increased cell death were observed. Nonetheless, the effects of longer exposure times and higher doses require further investigation. In addition, a clearer understanding of how autophagy and PANoptosis develop over time in ischemic skin flaps would help clarify their roles in flap ischemia and guide more precise modulation. This will require further studies.

Small RNA sequencing revealed that 3,4-DC downregulated the expression of miR-107-3p, an autophagy-suppressing miRNA. Functional assays confirmed that elevated miR-107-3p inhibited Wnt3a expression, which in turn activated mTOR and blocked TFEB translocation. Conversely, 3,4-DC-induced suppression of miR-107-3p restored Wnt3a activity, inhibited mTOR, and activated TFEB. The therapeutic effect was abrogated by Wnt3a inhibition or mTOR activation, confirming the regulatory axis. Although this study focused on miR-107-3p, we acknowledge that broader analysis of small RNA sequencing data – such as identifying additional miRNA candidates and performing pathway enrichment – could reveal parallel or compensatory regulatory networks involved in autophagy and PANoptosis. We consider this a valuable direction for future investigations.

However, before clinical application, safety and feasibility issues must be addressed. Although 3,4-DC has shown efficacy in preclinical settings and short-term safety as a CRM, its long-term toxicity remains unclear. Chronic use may pose risks such as hepatotoxicity, metabolic imbalance, or immune modulation, especially if it affects systemic autophagy beyond the local surgical site. Additionally, interspecies metabolic differences may alter its bioavailability and toxicity in humans. To bridge this gap, future studies should include pharmacokinetic and toxicological assessments in large animal models. It is also essential to explore local delivery methods (e.g., hydrogels, microneedle patches, and biodegradable scaffolds) to minimize systemic exposure while maximizing local efficacy. Similarly, the lack of pharmacokinetic and tissue distribution data for 3,4-DC limits the understanding of its safety and efficacy in ischemic skin, and its absorption, distribution, metabolism, and excretion characteristics and dermatologic applications remain unclear. We plan to address this issue in future studies to better guide dosing and safety.

Notably, 3,4-DC was administered continuously from surgery throughout the postoperative period, covering the critical window of necrosis development. Nevertheless, we acknowledge that time-course studies with various initiation and withdrawal points are valuable for clarifying the temporal dynamics of 3,4-DC. Owing to current budget constraints, this was beyond the scope of our study but remains an important direction for future research. In clinical practice, flap survival is usually improved by methods such as VEGF-based proangiogenic therapy, surgical delay, or ischemic preconditioning. These strategies mainly act by increasing perfusion, whereas 3,4-DC works by improving the ischemic microenvironment through reducing inflammatory cell death and enhancing autophagy. 3,4-DC may therefore be used as an adjunct rather than a replacement, and whether its combination with VEGF or preconditioning can further improve flap outcomes needs to be tested in future studies.

Importantly, 3,4-DC conferred protective effects in *db/db* diabetic mice, which are prone to flap failure due to poor perfusion and impaired healing. However, the rescue effect was only partial. In this model, 3,4-DC reversed surgery-induced weight loss, increased autophagy, suppressed PANoptosis, and improved flap viability, suggesting broader translational relevance. This highlights the challenges of treating ischemic complications in patients with diabetes and indicates the need for further optimization of therapeutic strategies in this context.

In general, despite its promise, several limitations should be noted. First, the long-term safety of 3,4-DC remains to be established; short-term toxicity studies are lacking, and extended studies are needed. Second, the contributions of other miRNAs or parallel signaling cascades have not been fully explored. Third, findings in mice may not be directly extrapolated to humans, warranting further validation in clinical studies. Finally, future research should examine the role of 3,4-DC in chronic ischemic settings and in elderly populations where flap healing is impaired.

## Conclusion

**Figure 3.**
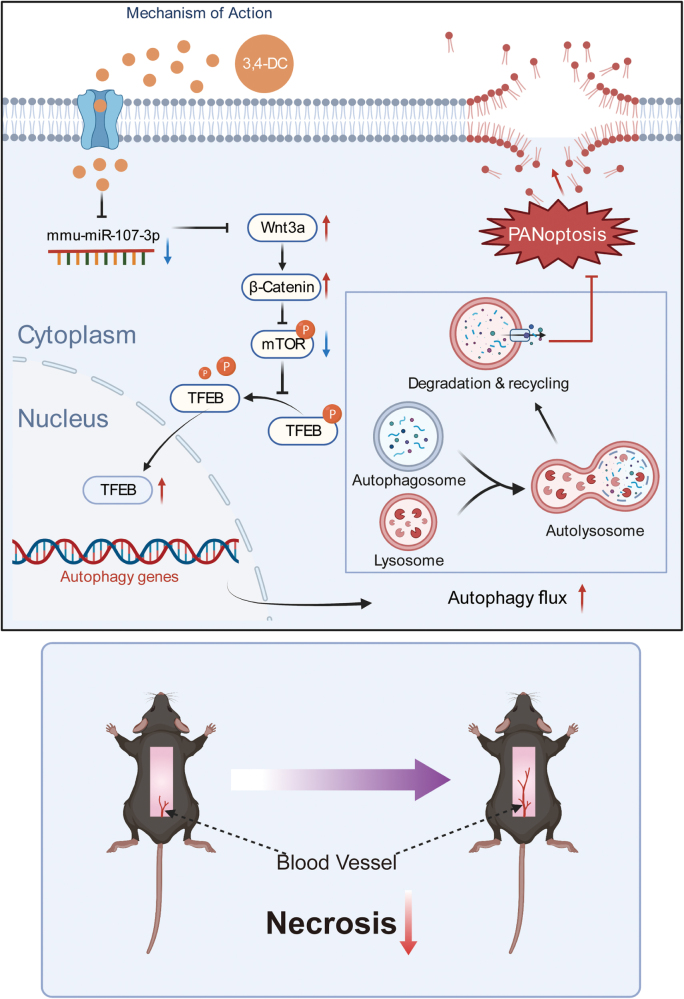
Schematic illustration of the proposed molecular mechanism of 3,4-DC, highlighting the roles of the miR-107-3p/Wnt3a/mTOR/TFEB signaling pathway, PANoptosis, and autophagy in the treatment of skin flap necrosis.

3,4-DC promotes ischemic flap survival by activating autophagy and inhibiting PANoptosis via the miR-107-3p/Wnt3a/mTOR/TFEB signaling pathway (Fig. 3). This effect is also partially reflected in the diabetic model, positioning 3,4-DC as a promising candidate for therapeutic intervention in ischemic and diabetic flap necrosis. However, further optimization is needed in diabetic models. Further studies are warranted to confirm its efficacy and safety in clinical settings.

## Data Availability

Additional data collected during this study are available from the corresponding author upon reasonable request.
